# Cardiopulmonary Arrest and Pulmonary Hypertension in an Infant with Pertussis Case Report

**DOI:** 10.1155/2021/6686185

**Published:** 2021-03-10

**Authors:** Patrick Ovie Fueta, Harry Onoriode Eyituoyo, Oghogho Igbinoba, Jon Roberts

**Affiliations:** ^1^Driscoll Children's Hospital, Corpus Christi, TX, USA; ^2^Mercer University School of Medicine, Macon, GA, USA

## Abstract

Pertussis is a vaccine-preventable disease with an incidence that has been trending upwards in the United States over the last two decades. This is evident by an increase in the incidence from 10,100 cases in 1974 to a peak of >48,000 cases noted in the last decade. Pertussis disease severity ranges from mild to severe, with resultant complications capable of causing significant morbidity and mortality. We report a case of pertussis in a 5-week-old female infant who presented with fever, paroxysms of cough, apnea, and seizures leading to cardiopulmonary arrest. Cardiopulmonary resuscitation lasted 11 minutes before the return of spontaneous circulation. She was transferred to our tertiary facility and admitted to the pediatric intensive care unit. Complete blood count revealed significant leukocytosis, chest X-ray revealed bilateral pulmonary edema with pleural effusion, and echocardiogram demonstrated pulmonary hypertension. Bordetella pertussis infection was confirmed on respiratory polymerase chain reaction. She was treated with antibiotics, ventilatory management, and other supportive care. She was discharged on room air after a hospital course of 7 weeks with care coordination between her primary care provider, pulmonologist, and neurologist. Despite the positive outcome in this case, it is important to note that managing severe pertussis involves multidisciplinary care, and the morbidity and cost implications can be mitigated on a population scale through vaccine optimization strategies.

## 1. Introduction

Pertussis is a respiratory infection caused by Bordetella pertussis, a Gram-negative bacillus. It produces various toxins and antigens that attack respiratory cells, causing inflammation and ciliary paralysis. It spreads via respiratory droplets and is highly infectious; one infected household contact can infect up to 80% of a household [1].

Prior to the development of the pertussis vaccine in the 1940s, there was a high incidence in the US with over 250,000 cases reported in 1934 [1, 2]. Postvaccine implementation, there was a significant reduction in incidence with numbers reaching a nadir of 10,010 in 1976. However, since the 1980s, a steady rise in incidence has been noted with the most recent peak documented in 2012: >48,000 cases, and 18 deaths were reported [1–3].

Common complications seen in the infant populations include apnea, seizures, respiratory distress, pulmonary hypertension, shock, and death. The highest rate of morbidity and mortality is among infants <6 months of age, presumably due to unattained complete vaccination series or nonvaccination [4]. Of all identified reservoirs of infection, undetected maternal infection is the most common accounting for approximately 33% of all cases [5].

In an effort to address the burden of disease from infant pertussis, the Advisory Council of Immunization Practices to the Centers of Disease Control and Prevention (CDC) in 2006 recommended “cocooning.” Cocooning involves in-hospital vaccination of postpartum mothers and adult household contacts of newborn infants with tetanus toxoid, diphtheria toxoid, and acellular pertussis vaccine (Tdap) prior to hospital discharge [4]. Maternal vaccination in the third trimester with Tdap has also been demonstrated to be an effective strategy in preventing infant pertussis [5]. Currently, the combined strategy of cocooning and maternal vaccination with Tdap in the third trimester has been identified as the most effective in preventing infantile pertussis infection [5, 6]. Severe pertussis infection can have significant burden on a personal, institutional, and statewide level especially during an epidemic, and the management requires coordinated multidisciplinary care.

We report the case of pertussis in a 5-week-old female infant who required prolonged hospitalization (7 weeks) after developing cardiopulmonary arrest, pulmonary hypertension, and postextubation atelectasis. In this case report, we highlight the importance of multidisciplinary care in the management of severe pertussis and the therapeutics needed for a favorable outcome.

## 2. Case Presentation

A 5-week-old female infant born at term with normal perinatal history was transferred from an outside facility with 4-day history of paroxysms of cough and a 1-day history of fever and seizure lasting <1 minute followed by apnea and cardiopulmonary arrest. Cardiopulmonary resuscitation (CPR) initiated by emergency medical services lasted for 11 minutes before return of spontaneous circulation was achieved. She was later stabilized by the outside hospital, transferred to our tertiary facility, and admitted to the pediatric intensive care unit (PICU). Further history obtained revealed a household contact (aunt) had cough. Her mother denies intrapartum pertussis vaccination and household participation in cocooning postpartum.

**Figure 1:**
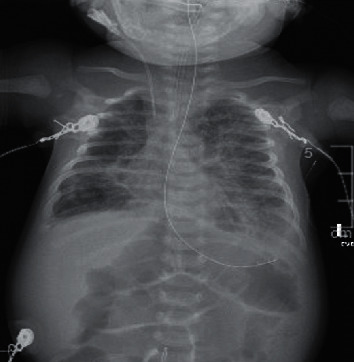
Initial chest X-ray showing bilateral pulmonary edema with left pleural effusion.

On physical examination in the PICU, she was mechanically ventilated and toxic appearing. Initial vital signs revealed a temperature of 36.8°C, blood pressure of 87/57 mmHg, heart rate of 175 beats per minute, respiratory rate of 30 cycles per minute, and SpO_2_ 100% (receiving 30% fraction of inspired oxygen (FiO_2_)). Complete blood count revealed significant leukocytosis (white blood cell count (WBC) of 76,000 *μ*L) treated with one round of exchange blood transfusion (EBT). Respiratory virus polymerase chain reaction (RVPCR) and sepsis workup were ordered, and the patient was treated empirically with azithromycin 10 mg/kg daily, cefepime 50 mg IV every 12 hours, and micafungin 10 mg/kg daily. Due to the history of seizure, she was started on intravenous (IV) levetiracetam 15 mg/kg every 12 hours. RVPCR was positive for Bordetella pertussis, Moraxella catarrhalis, and Rhino/enterovirus. Cefepime and micafungin were discontinued after blood cultures were negative for organism growth. Initial chest X-ray (CXR) revealed bilateral pulmonary edema with left pleural effusion (Figure 1).

**Figure 2:**
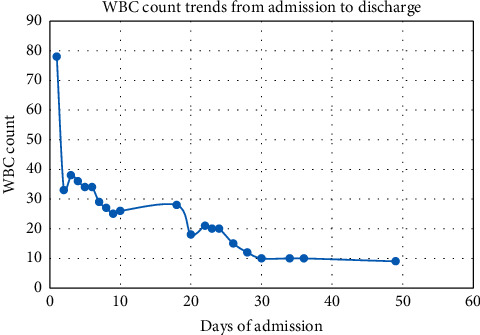
White blood cell trend postexchange blood transfusion.

Echocardiogram demonstrated pulmonary hypertension. The patient was placed on sildenafil 0.5 mg/kg every 6 hours, and aggressive diuresis was initiated with furosemide 1 mg/kg twice daily, chlorothiazide 10 mg/kg twice daily, and spironolactone 1 mg/kg twice daily. On day 22, she completed her antibiotics course, was extubated, and transitioned to oxygen via high flow nasal cannula. WBC trending showed the resolution of leukocytosis (Figure 2).

**Figure 3:**
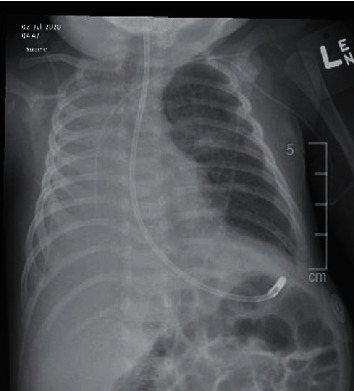
Chest X-ray showing new right lung atelectasis.

Postextubation, physical examination revealed decreased air entry over the right upper and middle lung zones. Repeat CXR revealed new right lung atelectasis with left retrocardiac atelectasis (Figure 3).

Treatment with aggressive pulmonary toilet was initiated using nebulized albuterol, 7% hypertonic saline, acetylcysteine 10% (100 mg/10 mL) 4 mL every 6 hours, and chest physiotherapy with high-frequency chest wall oscillation vest every 6 hours. CXR the following day revealed marked resolution of the atelectasis. She remained on increased oxygen support for 13 days after which she was weaned to oxygen via nasal cannula at 4 liter per minute. Upon completion of diazepam wean, electroencephalogram and magnetic resonance imaging demonstrated no abnormalities. Sildenafil was discontinued, and she was transferred from the PICU to one of the medical floors for continued management.

**Figure 4:**
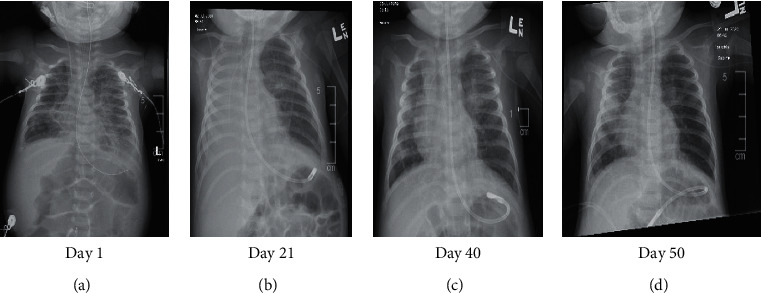
Radiologic trend showing progression of clinical condition from admission.

On day 48, she was weaned to room air, which she tolerated well. Repeat echocardiogram demonstrated resolution of the pulmonary hypertension. On day 50, her clinical state was markedly improved and CXR demonstrated continued improvement in atelectasis (Figure 4). The patient was discharged on room air after 7 weeks of hospitalization with close follow-up scheduled with her primary care provider, pulmonologist, and neurologist.

She was followed up by the pulmonology service one week after discharge via a telehealth visit. She continued to exhibit mild intermittent cough; however, she remained clinically stable. She continued pulmonary toilet regimen 4 hours as needed with an in-person follow-up visit scheduled in another week. During her office visit, she continued to exhibit mild, intermittent cough; however, her mother stated that symptoms were improving. Her growth chart demonstrated weight gain, and her vital signs were within normal limits. On examination, her breath sounds were clear bilaterally with no abnormalities noted. She was clinically stable, pulmonary toilet was recommended as needed, and follow-up was scheduled by pulmonology.

## 3. Discussion

Severe leukocytosis is associated with poor outcome in infants hospitalized with pertussis, and current modalities (extracorporeal membrane oxygenation and EBT) are often aimed at reducing the number of leukocytes [7]. Pulmonary hypertension is an uncommon complication of pertussis, with its presence inferring poor outcomes in infants and children [8, 9]. Pertussis toxin (PT) has been implicated in the pathophysiology of pulmonary hypertension by increasing cyclic adenosine monophosphate production in pulmonary vascular endothelial cells and inhibiting nitric oxide release, thereby resulting in pulmonary vasoconstriction [8]. Leukocyte thrombi in pulmonary veins have also been demonstrated to cause pulmonary hypertension; however, the exact pathophysiology is not well-understood [9].

Our patient presented with severe leukocytosis at the onset of her illness. In this case, the infant's high WBC count prompted early initiation of one round of EBT and supportive care. Death in young infants with pertussis is nearly always associated with extreme leukocytosis with lymphocytosis [7, 9]. It has been suggested that these deaths are due to aggregates of leukocytes in the small vessels of the lungs leading to intractable pulmonary hypertension. Several studies have also shown a high mortality rate in infants <6 weeks of age with pertussis and pulmonary hypertension [8]. Halasa et al. reported 4 fatal cases of pertussis in infants <9 weeks of age who developed pneumonia, refractory pulmonary hypertension, and respiratory failure and succumbed to their illness; they failed conventional methods of oxygenation, and pulmonary hypertension was resistant to nitric oxide and ECMO [7]. In contrast, our patient's pulmonary hypertension was successfully managed with CPR, mechanical ventilation, supportive care, and sildenafil therapy.

Our patient suffered a severe clinical course with seizures, apnea, and atelectasis after extubation, requiring anticonvulsant therapy, continued oxygenation, and prolonged PICU and hospital stay (34 days and 50 days, respectively). Furthermore, our patient had coinfection with Moraxella catarrhalis and rhino/enterovirus, and it is unclear if this may have contributed to the severity of her disease which poses potential limitations on our reported pertussis case. Fortunately, the patient recovered completely without obvious neurological sequelae or organ failure. Our case demonstrates that an infant with multiple poor prognostic predictors for survival (<1 year of age and pulmonary hypertension) can survive with a multidisciplinary approach to management. However, it should be emphasized that there is an increased cost of care associated with admissions to the PICU and prolonged length of hospital stay [10]. Thus, prevention and early diagnosis/intervention are essential to improving disease outcomes and reducing the healthcare burden associated with managing pertussis.

In this case of severe infant pertussis, cocooning was not done. The source of the infection may have been the patient's household contact who had a history of cough. This emphasizes the importance of disease surveillance and revaccination to protect vulnerable populations. Currently, the most effective measure in reducing infant pertussis incidence involves combining “cocooning” with maternal vaccination in the third trimester [1, 4]. Other proposed interventions to address the burden of infantile pertussis include vaccination of healthcare professionals and neonatal vaccination with a single dose of the acellular pertussis (aP) vaccine plus the hepatitis B vaccine at birth. In a recent study conducted by Wood et al., a significantly higher proportion of infants who received a single dose of the aP vaccine plus the hepatitis B vaccine at birth developed detectable antibodies to both PT and pertactin compared with a control group [11]. More research is required to demonstrate the safety of this strategy and possibly translate these results into vaccine recommendations that can contribute to addressing the burden of pertussis illness in the US, especially in situations where maternal vaccination was not initiated.

## 4. Conclusion

Pertussis is a vaccine-preventable disease with an upward trend in incidence and prevalence in the United States over the last two decades. Our case report highlights the severity of pertussis in infants too young to be vaccinated and discusses several complications of the disease. We emphasize the importance of implementing vaccination strategies to reduce the burden of illness among infants. Future pertussis vaccine studies should focus on the initiation of vaccination at birth, especially in infants without maternal vaccination.

## Data Availability

The data used to support the findings of this study are available on reasonable request from the corresponding author.

## Abbreviations:

CDC: Centers of Disease Control and Prevention
Tdap: Tetanus and diphtheria toxoids and acellular pertussis vaccine
CPR: Cardiopulmonary resuscitation
PICU: Pediatric intensive care unit
FiO_2_: Fraction of inspired oxygen
WBC: White blood cell count
EBT: Exchange blood transfusion
RVPCR: Respiratory virus polymerase chain reaction
IV: Intravenous
CXR: Chest X-ray
PT: Pertussis toxin
AP: Acellular pertussis.

## Additional Points

*Summary*. This case discusses the complications of severe infantile pertussis and emphasizes the importance of focused vaccination strategies in reducing pertussis morbidity and mortality among infants.
